# Combining isothermal recombinase polymerase amplification with lateral flow assay for diagnosis of *P*. *cynomolgi* malaria infection

**DOI:** 10.1371/journal.pntd.0011470

**Published:** 2023-07-05

**Authors:** Pongruj Rattaprasert, Chutima Chavananikul, Wirasak Fungfuang, Porntip Chavalitshewinkoon-Petmitr, Paviga Limudomporn

**Affiliations:** 1 Department of Protozoology, Faculty of Tropical Medicine, Mahidol University, Ratchawithi Road, Bangkok, Thailand; 2 Department of Zoology, Faculty of Science, Kasetsart University, Bangkok, Thailand; 3 Kasetsart University Research and Development Institute (KURDI), Kasetsart University, Bangkok, Thailand; Universidade Federal de Minas Gerais, BRAZIL

## Abstract

**Background:**

*Plasmodium cynomolgi* is a nonhuman primate parasite that causes malaria in humans and is transmitted by the *Anopheles* mosquito. Macaques, the natural hosts of *P*. *cynomolgi*, are widely distributed in Asia, especially in Southeast Asia. Anthropogenic land-use changes and wildlife habitat reduction due to local environmental changes, deforestation, urban expansion, and construction increased the frequency of human-macaque-vector interactions and facilitated the emergence of zoonotic malaria, causing an exponential increase in the infection rates in this area. Although microscopic tools are the gold standard for malaria diagnosis, they have very low sensitivity. Therefore, disease control and prevention require rapid, sensitive and accurate diagnostic tests.

**Methodology/Principle findings:**

This study aims to develop a diagnostic method using a recombinase polymerase amplification (RPA) combined with a lateral flow (LF) strip method to specifically diagnose *P*. *cynomolgi*. Laboratory validation determined the method’s sensitivity and specificity compared to the nested PCR method. The lower limit of detection was 22.14 copies/μl of recombinant plasmid per reaction. The combination method represented 81.82% sensitivity and 94.74% specificity compared to the nested PCR.

**Conclusions/Significance:**

The diagnostic testing developed in this study combines a recombinase polymerase amplification (RPA) and a lateral flow (LF) strip, offering rapid high sensitivity and specificity. Further development of this technique could make it a promising method for detecting *P*. *cynomolgi*.

## Author summary

*P*. *cynolmogi* is zoonotic protozoa that cause malaria in both macaques and humans. In the past few years, the human infection rate from this parasite has risen throughout Southeast Asia. We have developed molecular testing for diagnosing *P*. *cynolmogi* based on a recombinase polymerase amplification (RPA) combined with a lateral flow (LF) strip. The rapid, sensitive, and portable of our testing represents a promising assay for the *P*. *cynomolgi* diagnosis method.

## Introduction

Malaria is a hemoparasitic disease in humans and nonhumans transmitted via the infected female *Anopheles* mosquito. It is caused by over 200 species of protozoa from the genus *Plasmodium*. Four *Plasmodium* species, *Plasmodium ovale (P*. *ovale)*, *P*. *vivax*, *P*. *malariae*, and *P*. *falciparum*, are the leading cause of malaria in humans. However, numerous simian *Plasmodium* species have been reported to infect humans naturally. Among these, *P*. *cynomolgi*, a predominant malaria parasite found in Southeast Asia Old-World monkeys, naturally causes zoonotic infections in humans [1,2]. The definitive hosts of *P*. *cynomolgi* are long-tailed and pig-tailed macaques (*Macaca fascicularis* and *Macaca nemestrina*, respectively), commonly found in many Southeast Asian countries [3]. After the first report of natural *P*. *cynomolgi* human infection in 2011 [1], the infection rate has risen throughout Southeast Asia, with the global prevalence ranging from 0 to 1.4% in humans [4]. Land-use change, including deforestation, rapid population growth, and urban area expansion, brings humans and wildlife closer, increasing infection risks via vector-host-pathogen interactions [5].

As nearly all patients infected with *P*. *cynomolgi* are asymptomatic, highly sensitive diagnostics are required for disease control and prevention in malaria-endemic areas [6,7]. Parasites from asymptomatic carriers are more infectious to the *Anopheles* mosquito and a significant source of gametocytes for local mosquito vectors, increasing the rate of malaria transmission. As the morphological and biological characteristics of *P*. *cynomolgi* are similar to those of *P*. *vivax*, misdiagnosis is highly likely with microscopic examination, the gold standard for malaria diagnosis [8–11]. Moreover, the accuracy of the microscopic diagnosis depends on the level of malaria parasitemia and the examiner’s skills [12,13]. Recently, molecular testing using polymerase chain reaction (PCR) and other nucleic acid amplification methods has proven more sensitive and specific in detecting parasites. However, these techniques are time-consuming, and require skilled technicians and expensive equipment [14,15].

RPA is an isothermal nucleic acid amplification method ideal for limited resource areas due to its low-resource requirements. This system requires a combination of three enzymes: polymerases, single-stranded DNA-binding proteins, and recombinases to amplify the target DNA sequences at a constant temperature [16]. The DNA amplicons can be detected by agarose gel electrophoresis (AGE), real-time fluorescence, or a lateral flow dipstick (LFD). Detection using LFD makes this method ideal for a quick readout, which is preferred at the point of need, especially in low-resource settings.

In this study, we developed a method combining RPA and LFD to detect *P*. *cynomolgi*. Specific primers were designed using the sexual *18sRNA* gene as a template or amplification and the LF strip for the readout. We evaluated the specificity and sensitivity of this assay by testing it on macaque blood samples.

## Materials and methods

### Ethics statement

This research was approved by the Department of National Parks, Wildlife and Plant Conservation, Thailand, for study in a protected area, collection of the blood samples and released of wild macaques (Permit Number: 0909.204/14187). All procedures were carried out in accordance with the Guide for the Care and Use of Laboratory Animals of the National Institutes of Health, U.S.A., and were approved by the Animal use and care committee of Kasetsart University Research and Development Institute, Kasetsart University, Thailand (ID: ACKU59-SCI-011).

### Design of RPA primers

A set of RPA primers was designed by the NCBI Primer-BLAST website based on the sexual stage-small subunit ribosomal RNA gene of *P*. *cynomolgi* (GenBank Acc. No. AB287288). The database was set as RefSeq representative genomes. The product and primer sizes were set as 120–300 bp and 30–35 nt, respectively. Other parameters were set as default. The primers with target-specific labels were tagged with Biotin and Fluorescein-5-isothiocyanate (FITC) (Table 1) and synthesized by Macrogen, Inc. (Seoul, Republic of Korea).

**Table 1:** Sequences of sexual stage-small subunit ribosomal RNA gene of *Plasmodium cynomolgi* recombinase polymerase amplification (RPA) primers.

Primer name	Sequence (5’–3’)	Amplicon size (bp)
PC_AB287288_1425F	Biotin-GATATGTATGATTTGCTAAATTGCGGTCGC	144
PC_AB287288_1569R	FITC-GGTATGATAAGCCAGGGAAGTGAAGTCCAGGGAAC	

### Sample collection and DNA template preparation

#### DNA samples

The genomic DNA (gDNA) from human malarial parasites, *P*. *falciparum*, *P*. *vivax*, *P*. *malariae*, *P*. *ovale*, and *P*. *knowlesi*, used as specificity controls, were provided by Dr. Porntip Chavalitshewinkoon-Petmitr of the Department of Protozoology, Faculty of Tropical Medicine, Mahidol University.

#### Macaque blood samples

Ninety-three blood samples were collected from wild macaques by Dr. Wirasak Fungfuang in three areas of Thailand from July 2017–July 2019, including samples from wild *M*. *arctoides* at the Pa La U waterfall, Huahin district, Prachuap Kiri Khan Province, wild *M*. *leonina* at the Khao Yai National Park, Nakornratchasima Province, and wild *M*. *fascicularis* at Chang Island, Mu Ko Ranong National Park, Ranong Province. The gDNA was extracted directly from samples using GeneJET Whole Blood Genomic DNA Purification Mini Kit (Thermo Fisher Scientific, USA) and stored at −20°C until use. In our previous study, all gDNA samples showed negative results for dengue virus (DENV), Zika virus (ZIKV), Chikungunya virus (CHIKV), *Leptospira* spp., and *Burkholderia pseudomallei* using molecular detection [17,18]. To analyze *Plasmodium* species, a nested PCR approach was employed, following the previously described method [19]. *Plasmodium*-positive samples underwent further examination using species-specific primers to identify *P*. *knowlesi*, *P*. *coatneyi*, *P*. *cynomolgi*, *P*. *inui*, and *P*. *fieldi*, as outlined in a previous study [20] (Table 2) and *P*. *cynomolgi* RPA-LFD assay.

**Table 2:** Macaque blood samples characteristics and results of malaria screening by nested PCR.

	*n*	Percentage
**Macaque Species**		
*Macaca arctoides*	32	34.41
*Macaca fascicularis*	36	38.71
*Macaca leonina*	25	26.88
**Macaque blood samples screening by nested PCR**		
*Plasmodium*-negative	63	67.74
*Plasmodium*-positive	30	32.26
Single infection		
*P*. *cynomolgi*	6	6.45
Non *P*. *cynomolgi*	10	10.75
Mix infection		
*P*. *cynomolgi* with others	5	5.38
Non *P*. *cynomolgi* with others	4	4.3
Unidentified species	5	5.38

### Generation of plasmid DNAs

DNA was amplified using the gDNA of *P*. *cynomolgi* samples and *P*. *inui* macaque blood samples. The amplification reaction mixture contained 1× Phusion HF Buffer, 0.2 mM deoxynucleoside triphosphates (dNTPs), and 0.5 μM of each *Plasmodium* spp. (rPLU1, rPLU5) primer [14]. Reactions were performed using a 20 μL reaction mixture containing 1 μL genomic DNA and 0.4 U Phusion High–Fidelity DNA Polymerase (Thermo Fisher Scientific, USA). The cycling conditions were as follows: 98°C for 30s, 35 cycles at 98°C for 10 s, 52°C for 10 s, and 72°C for 45 s and a final extension at 72°C for 5 min. The PCR amplicons were confirmed using AGE on a 1.4% agarose gel and staining with RedSafe Nucleic Acid Staining Solution (iNtRON Biotechnology, Korea). The PCR fragments were purified using a QIAquick PCR purification kit (Qiagen, Valenica CA, USA) and then cloned into pMiniT 2.0 vector using the NEB PCR Cloning Kit (New England Biolabs, USA) following the manufacturer’s recommendations. Colony PCR was performed using Taq DNA Polymerase with Standard Taq Buffer (New England Biolabs, USA) and primers corresponding to those used to amplify the cloned PCR fragment to verify successful cloning. The PCR fragments were analyzed on an agarose gel to ascertain the correct PCR fragments. The clones with correct PCR fragments were further purified from overnight cultures using FavorPrep Plasmid Extraction Mini Kit (Favorgen, Taiwan) following the manufacturer’s recommendations and were delivered for sequencing (Celemics, Seoul, Korea). The concentration and purity of the purified plasmid DNA were measured using NanoDrop 2000 (Thermo Fisher Scientific Inc., USA) following the manufacturer’s instructions. The plasmid DNA Genomic Equivalence was calculated using the following equation:

(Xg/μLDNA/[transcriptlengthnucleotide×660])×6.022×1023=Ymolecule/μL

### RPA assay

RPA was performed in a 50 μL reaction mixture using the TwistAmp basic kit (TwistDx, Inc., Maidenhead, United Kingdom) according to the manufacturer’s instructions. All the reaction components (0.05 μM RPA primers, 1 × rehydration buffer, and DNase-free water), except DNA templates and 14 mM magnesium acetate, were prepared as a master mix distributed into a 0.2 mL Eppendorf tube strip containing a dried enzyme pellet. Then, after adding 5 μL of each DNA template to the mixture aliquots, 2.5 μL of magnesium acetate was pipetted into the tube lids and centrifuged into the reaction mix using a benchtop centrifuge. The reaction tubes were immediately incubated at 39°C for 20 min on a heating block.

### LFD assay

The RPA-amplified products were visualized based on the calorimetric signals on the nucleic acid LFD strips, which were fabricated by Kestrel BioSciences Thailand Co. Ltd. Briefly, 10 μL of RPA-amplified products were mixed with 90 μL of running buffer (phosphate-buffered saline and Tween 20) in a fresh Eppendorf tube. Subsequently, the LFD strip was immersed into the mixture and incubated for 2 min at room temperature. The solution migrated along the LFD strip through the conjugate pad coated with gold nanoparticles labeled with anti-FITC. The complexes were captured by the antibiotin embedded on the test line and goat antimouse IgG on the control line. After 2 min, the strip with one visible line in the control area was considered negative, while the strip with two visible lines in both control and test areas was considered positive.

### Analytical limit of detection (LOD) and cross-reactivity testing of the RPA-LFD assay

To evaluate the specificity of the assay, gDNA from patients infected with five human *Plasmodium* species (*P*. *falciparum*, *P*. *vivax*, *P*. *malariae*, *P*. *ovale*, and *P*. *knowlesi*) and the *P*. *cynomolgi* and *P*. *inui* plasmid DNA were used as templates for detection. A DNA-free template was included as a negative control. All amplicons were evaluated using AGE and LFD, and the results were compared.

To set the sensitivity and the lower detection limit of the RPA-LFD assay, we used ten-fold serial dilutions (22.14 × 10^6^–22.14 × 10^−1^ copies/μL) of *P*. *cynomolgi* plasmid DNA as templates for RPA under the same conditions specified before. All RPA-amplified products were screened by AGE and LFD, and the results were compared. Meanwhile, quantitative PCR (qPCR) with the same primers and templates were performed as the control for RPA.

The qPCR was carried out in a 20 μl reaction, consisting of 10 μl Luna Universal qPCR Master Mix (New England Biolabs, USA), 0.5 μl of each primer (10 μM), 1 μl of plasmid DNA and 8 μl of nuclease-free water. The reactions were conducted in triplicate on CFX96 Touch Real-Time PCR Detection System (Bio-Rad, Hercules, CA, USA). The thermal cycling program included an initial denaturation step at 95°C for 60 seconds, followed by 45 cycles of denaturation at 95°C for 15 seconds and annealing/extension at 60°C for 30 seconds. To serve as a negative control, DNase-free water was used as a non-template control in all reactions. A sample was considered positive if the qPCR threshold cycle (CT) value of the plasmid DNA crossed the zero baseline before the 40th cycle. After the 45^th^ cycle, the melting curve analyses were performed between 60°C and 95°C to assess the quality of the final PCR products.

### Evaluation of the RPA-LFD assay using macaque blood samples

**Fig 1:**
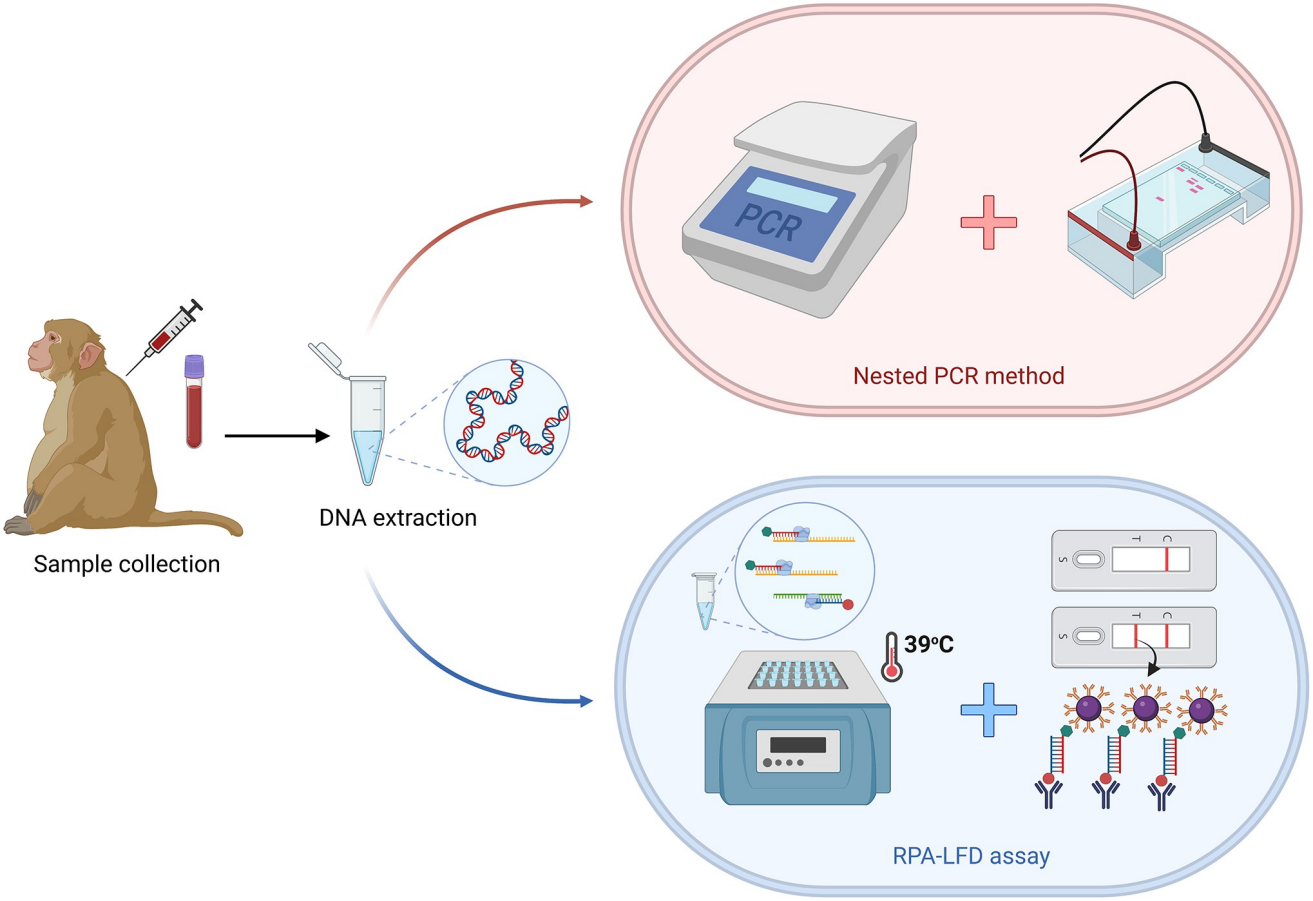
Workflow of the RPA-LFD assay and nested PCR method using malaria-positive samples. The illustration was created with BioRender.com.

Thirty *Plasmodium*-positive blood samples from wild macaques determined by nested PCR were used to assess the performance of the RPA-LFD assay. Two hundred μL of the blood samples, kept in sterile containers, was immediately used to extract gDNA using the GeneJET Whole Blood Genomic DNA Purification Mini Kit (Thermo Fisher Scientific, USA). RPA was performed, as previously described, using 5 μL of DNA for each reaction. The RPA-amplified products were screened by LFD, and the results were compared with the nested PCR using species-specific primers to detect *P*. *cynomolgi* as described previously [20]. For each assay, a positive control (plasmid DNA of *P*. *cynomolgi*) and a negative control (DNA-free template) were included to rule out false positives or false negatives (Fig 1).

### Statistical analysis

Analysis of data was performed using GraphPad Prism 9. Kappa statistics were used to assess the diagnostic performance of the RPA-LFD assay compared to nested PCR (considered here as the reference assay).

## Results

### Specificity of the RPA-LFD assay for detecting *P*. *cynomolgi*

**Fig 2:**
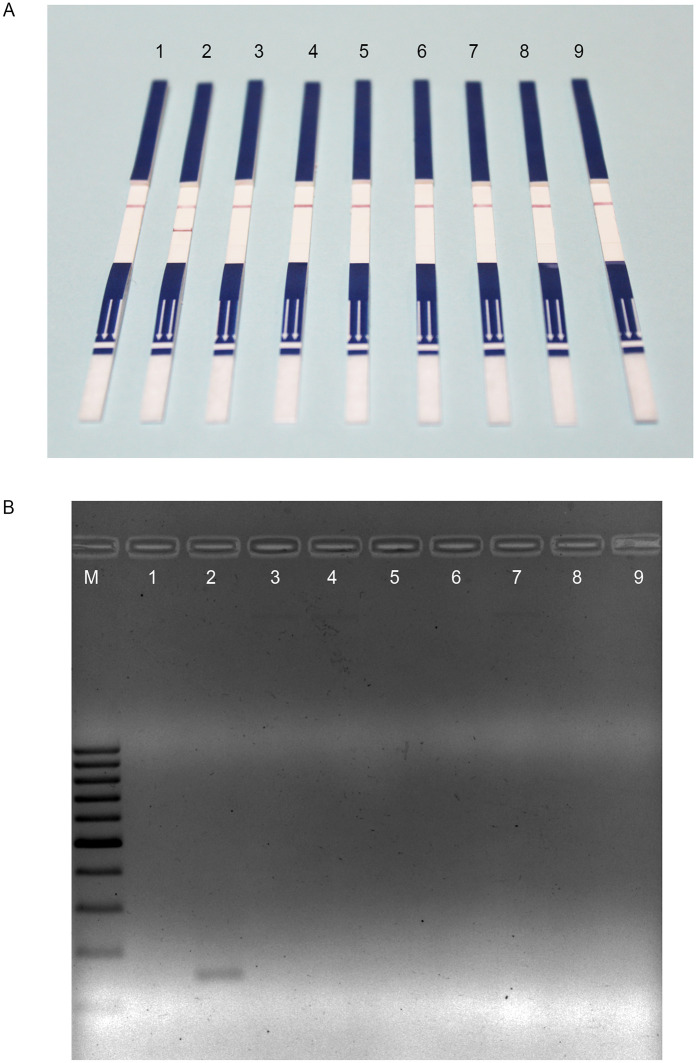
Specificity of the RPA-LFD assay for detecting *Plasmodium cynomolgi*. (A) LFD assay for RPA-amplified products at 39°C for 20 min from different *Plasmodium* species. Strips 1 and 2 contain the DNA-free template and 1 ng of *P*. *cynomolgi* plasmid DNA, respectively. Strips 3–7 consist of 5 ng genomic DNA of *P*. *falciparum*, *P*. *vivax*, *P*. *malariae*, *P*. *ovale*, and *P*. *knowlesi*, respectively. Strips 8 and 9 contain 1 ng of *P*. *inui* asexual and sexual-type recombinant plasmid DNA. (B) AGE results for the identical RPA amplicons amplified under the same conditions after purification. Lane M is a 100 bp molecular ladder (Thermo Fisher Scientific Inc., USA).

We evaluated the ability of the RPA-LFD assay to distinguish between *P*. *cynomolgi* and other *Plasmodium* species. The assay showed specificity to *P*. *cynomolgi* DNA without any cross-reactivity with other *Plasmodium* species (Fig 2). The LFD assay gave only a clear reddish–purple line in the test area (positive reaction) from the RPA-amplified products of *P*. *cynomolgi* (Fig 2A), as shown by the expected 144 bp amplicon on AGE after purification (Fig 2B). Contrastingly, no LFD test lines and AGE bands were observed after the amplification of gDNA templates from other *Plasmodium* species.

### LOD of RPA-LFD assay for detecting *P*. *cynomolgi*

**Fig 3:**
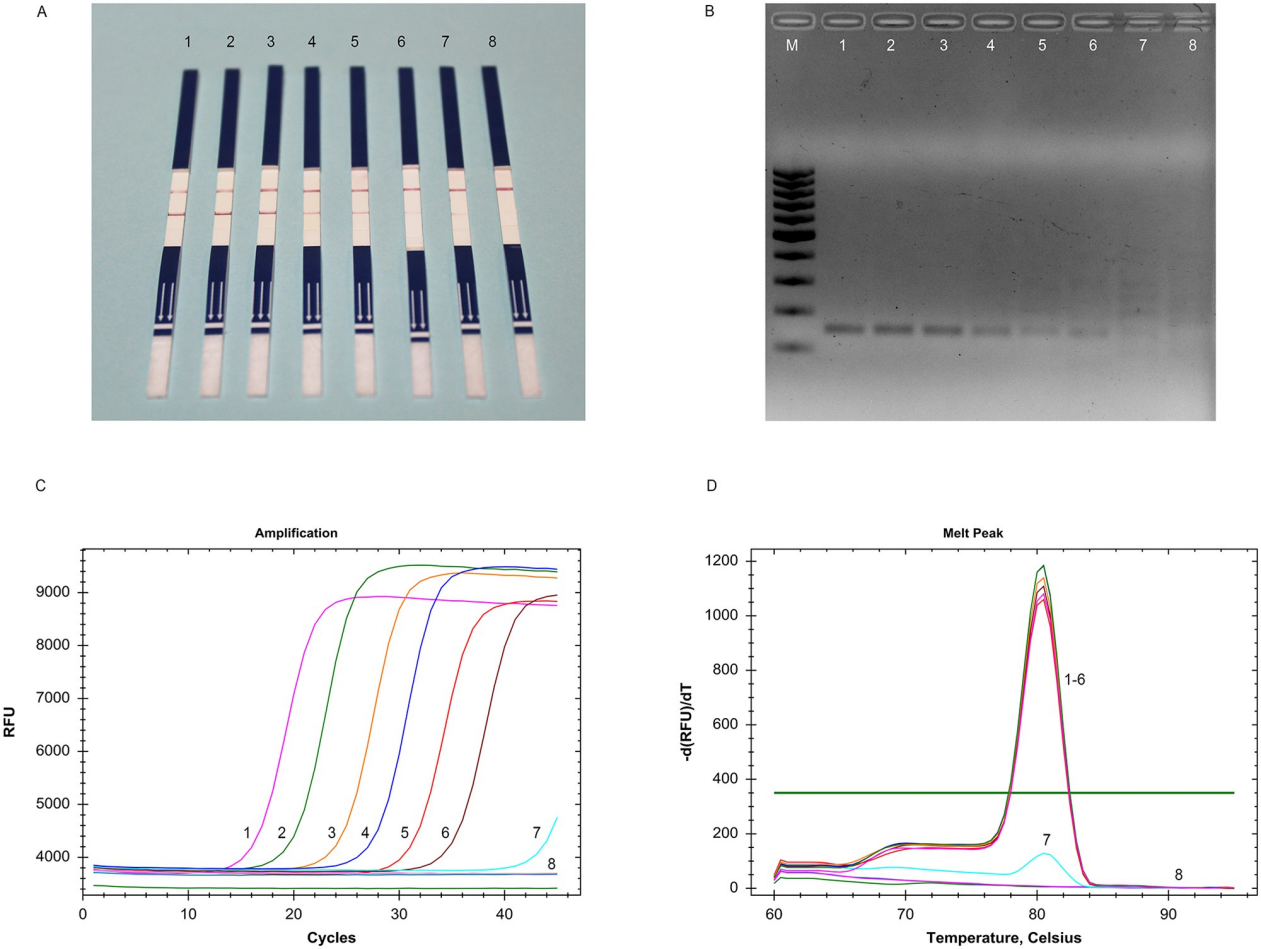
LOD of the RPA-LFD assay to ten-fold serial dilutions of *Plasmodium cynomolgi* plasmid DNA. (A) LFD assay for RPA-amplified products at 39°C for 20 min. Strips 1–8 correspond to *P*. *cynomolgi* plasmid DNA concentrations of 22.14 × 10^6^, 22.14 × 10^5^, 22.14 × 10^4^, 22.14 × 10^3^, 22.14 × 10^2^, 22.14 × 10, 22.14 and 11.07 copies/μL, respectively. (B) AGE results for the same RPA amplicons amplified under the same conditions after purification. Lanes M is a 100 bp molecular ladder (Thermo Fisher Scientific Inc., USA). (C) qPCR results for *P*. *cynomolgi* plasmid DNA concentrations of 22.14 × 10^6^, 22.14 × 10^5^, 22.14 × 10^4^, 22.14 × 10^3^, 22.14 × 10^2^, 22.14 × 10, 22.14 and 11.07 copies/μL, respectively. (D) Melting curve analysis of qPCR amplicons.

The LOD was defined as the minimum concentration of DNA at which a positive signal is observed by RPA-LFD assay. In this study, a known copy number of recombinant plasmid DNA of *P*. *cynomolgi* was serially diluted and used in RPA and qPCR assay. Each dilution was tested three times independently. The result showed that our RPA-LFD assay was highly sensitive to *P*. *cynomolgi* with a detection limit of 22.14 copies/μL of recombinant plasmid per reaction (10^−7^ dilution) (Fig 3A), which was ten times greater than that of RPA-AGE assay (22.14 × 10 copies/μL) (Fig 3B) and qPCR (Fig 3C). The identity of the PCR products was confirmed through the melting curve analysis of the qPCR amplicons, which showed a distinct single peak (Fig 3D).

### Evaluation of RPA-LFD assay using blood samples of wild macaques

We tested 30 samples to evaluate the detection performance of the RPA-LFD assay. A comparison of the results obtained from the nested PCR and RPA-LFD assays grouped by the infection type is shown in Table 3. RPA-LFD assays showed positive results both in single and mixed infection. Of these, 11 samples (36.67%) tested positive for *P*. *cynomolgi* using the nested PCR method, while the RPA-LFD assay detected nine positive samples (30%) with two false negatives, making its sensitivity at 81.82%. Of the 19 samples indicated as negative by the nested PCR method, one tested as false positive by RPA-LFD assay, yielding a specificity of 94.74%. The Cohen’s kappa index of the RPA-LFD assay was 0.78, significantly consistent with the reference method (Table 4).

**Table 3:** Comparison of *P*. *cynomolgi* RPA-LFD assays detection in wild macaques’ blood samples to nested PCR.

Infection type	Nested PCR method	RPA-LFD assay
	Number of positive	Number of positive
Single infection		
* P*. *cynomolgi*	6	6
Non *P*. *cynomolgi*	0	1
Mix infection		
* P*. *cynomolgi* with others	5	3
Non *P*. *cynomolgi* with others	0	0
Unidentified species	0	0
Total	11	10

**Table 4:** The detection performance of RPA-LFD assay for *P*. *cynomolgi* detection compared with the nested PCR method in the wild macaques’ blood samples.

Method		Nested PCR method		Cohen’s kappa*	Sensitivity	Specificity
		Positive	Negative			
RPA-LFD assay	Positive	9	1	0.78	81.82%	94.74%
	Negative	2	18			

*The Cohen’s kappa coefficient was interpreted as = 0, No agreement; 0.01–0.20, slight agreement; 0.21–0.40, fair agreement; 0.41–0.60, moderate agreement; 0.61–0.80, substantial agreement; 0.81–1.00, almost perfect agreement.

## Discussion

Close human–animal-vector encounters because of land-use changes increase the epidemiological risk of developing zoonotic infectious diseases, including malaria [21–26]. The first *P*. *cynomolgi*-infected human was reported in 2011 [1]. In the following years, several reports emerged from Southeast Asia countries, where long-tailed macaques (*M*. *fascicularis*) and pig-tailed macaques (*M*. *nemestrina*), the natural definitive hosts, are found [3]. The accurate and rapid detection of *P*. *cynomolgi*-infected patients and animals is critical to prevent and manage this disease in the endemic regions [6].

Light microscopy-based techniques are the gold standard for malaria diagnosis. However, their accuracy and sensitivity depend on the examiner’s skill and the sample’s parasitemia levels. [12]. Moreover, this technique cannot reliably distinguish between *P*. *vivax* and *P*. *cynomolgi* [8–11]. Although molecular techniques such as PCR are highly sensitive and specific in diagnosing malaria, they are time-consuming and require experienced technicians and specialized expensive instruments [14, 15]. RPA is the isothermal amplification technique that can amplify specific DNA fragments at a single temperature, producing a specific product within 20 minutes using a few low-cost instruments [16]. This technique can be integrated with the LFD method to detect amplicons rapidly. Because of its simplicity, portability, high sensitivity, rapid detection time, low operating temperature, and cost, the RPA-LFD assay is the ideal diagnostic method for malaria.

To our knowledge, this is the first report showing the successful development of a *P*. *cynomolgi* RPA-LFD assay. We established the RPA-LFD system and evaluated its diagnostic validity using *P*. *cynomolgi* noninfectious and infectious blood samples. The sexual *18sRNA* gene RPA primers were designed following the RPA guidelines: 30–35 nucleotides in length, GC content between 40–51.4%, low potential for homo/heterodimers specific, and yield a 144 bp DNA product. The assay demonstrated high sensitivity (81.82%) and specificity (94.74%) compared to the nested PCR, which was used as the reference test. This assay could detect 22.14 copies/μl of recombinant plasmid per reaction. Our *P*. *cynomolgi* RPA-LFD could detect 3–6 parasites/μl, relying on the copy number of *18sRNA gene* per parasite varying from four to eight in *Plasmodium* species. [27] The test’s sensitivity is higher than the microscopic technique (limit of detection [LOD] = ~10 parasites/μl) in the controlled human malaria infection (CHMI) trial [28]. Evaluation of the specificity of the RPA-LFD assay using specific sexual *18sRNA* gene primers showed a lack of cross-reactivity with other *Plasmodium* species. These results reveal that the sensitivity and specificity of this system are ideal for *P*. *cynomolgi* diagnosis testing.

The complex and time-consuming sample preparation step for reducing DNA polymerase inhibitors is the limitation of this technique. The result should be interpreted immediately within 2 minutes to avoid false positive results due to contamination. Future work could design a suitable sample extraction and minimize the chance of contamination.

In conclusion, we developed the RPA-LFD system for the specific diagnosis of *P*. *cynomolgi* in simian blood samples. This method is a rapid, portable, and accurate tool for detecting *P*. *cynomolgi* infections. The result of this assay can be interpreted by the naked eye. Comparison of the results of this assay with that of nested PCR showed its high sensitivity and specificity. Based on these findings, this RPA-LFD assay is a potential diagnostic test for detecting *P*. *cynomolgi*.
